# Mitochondrial DNA deletions and neurodegeneration in multiple sclerosis

**DOI:** 10.1002/ana.22109

**Published:** 2011-03

**Authors:** Graham R Campbell, Iryna Ziabreva, Amy K Reeve, Kim J Krishnan, Richard Reynolds, Owen Howell, Hans Lassmann, Doug M Turnbull, Don J Mahad

**Affiliations:** 1 Institute of Ageing and Health, Mitochondrial Research Group, Newcastle UniversityNewcastle upon Tyne, UK; 2 Wolfson Neuroscience Laboratories, Imperial College Faculty of MedicineLondon, UK; 3 Department of neuroimmunology, Center for Brain Research, Medical University ViennaVienna, Austria

## Abstract

**Objective** Cerebral atrophy is a correlate of clinical progression in multiple sclerosis (MS). Mitochondria are now established to play a part in the pathogenesis of MS. Uniquely, mitochondria harbor their own mitochondrial DNA (mtDNA), essential for maintaining a healthy central nervous system. We explored mitochondrial respiratory chain activity and mtDNA deletions in single neurons from secondary progressive MS (SPMS) cases.

**Methods** Ninety-eight snap-frozen brain blocks from 13 SPMS cases together with complex IV/complex II histochemistry, immunohistochemistry, laser dissection microscopy, long-range and real-time PCR and sequencing were used to identify and analyze respiratory-deficient neurons devoid of complex IV and with complex II activity.

**Results** The density of respiratory-deficient neurons in SPMS was strikingly in excess of aged controls. The majority of respiratory-deficient neurons were located in layer VI and immediate subcortical white matter (WM) irrespective of lesions. Multiple deletions of mtDNA were apparent throughout the gray matter (GM) in MS. The respiratory-deficient neurons harbored high levels of clonally expanded mtDNA deletions at a single-cell level. Furthermore, there were neurons lacking mtDNA-encoded catalytic subunits of complex IV. mtDNA deletions sufficiently explained the biochemical defect in the majority of respiratory-deficient neurons.

**Interpretation** These findings provide evidence that neurons in MS are respiratory-deficient due to mtDNA deletions, which are extensive in GM and may be induced by inflammation. We propose induced multiple deletions of mtDNA as an important contributor to neurodegeneration in MS.

In the majority of patients, multiple sclerosis (MS) begins with a relapsing-remitting course followed by a gradual progression of irreversible neurological impairment (secondary progressive MS [SPMS]).^1^ Although the whole brain atrophies with advancing disease, reflecting loss of both neurons and axons, radiological studies with long-term follow-up show accelerated gray matter (GM) atrophy correlating with clinical disability during the progressive stage of MS.^2^, ^3^ Thus understanding the mechanisms of neurodegeneration is vital for identifying ways to intervene the progression of MS.^4^, ^5^

Mitochondrial defects are increasingly recognized to play a role in the pathogenesis of MS.^6–10^ Energy in the form of ATP is most efficiently produced by mitochondria, which also play a role in calcium handling, production of reactive oxygen species (ROS), and apoptosis.^11^ Uniquely mitochondria harbor their own DNA (mitochondrial DNA [mtDNA]), the only non-nuclear DNA in cells, which encodes 13 polypeptides featured in 4 of the 5 respiratory chain complexes.^12^ The importance of mtDNA for maintaining a healthy central nervous system (CNS) is highlighted by a number of primary mtDNA disorders, where the entirely nuclear DNA-encoded complex of mitochondrial respiratory chain, complex II, is spared.^13^, ^14^ The loss of complex IV (cytochrome c oxidase [COX]), the catalytic subunits of which are encoded by mtDNA, with intact complex II (respiratory deficiency) is considered a histochemical hallmark of biochemical defects in primary mtDNA disorders.^15^

Besides inherited defects, induced mtDNA mutations (deletions and point mutations) within neurons are well recognized in aging and neurodegenerative disorders.^16–18^ mtDNA is particularly vulnerable to oxidative damage due to its presence in a highly oxidative environment and lack of protective histones.^19^, ^20^ Processes that repair double-stranded breaks have been proposed as an important mechanism for the formation of mtDNA deletions, the predominant type of induced mtDNA mutations in neurodegeneration.^17^, ^18^, ^20–22^ As a single cell contains many copies of mtDNA, for a biochemical defect to manifest, the ratio between deleted to wild-type or healthy mtDNA (heteroplasmy) needs to exceed a certain threshold.^23^ The increase in heteroplasmy level is through a process of clonal expansion whereby 1 mutation becomes dominant within the cell.^12^, ^18^, ^21^, ^24^ Interestingly, different mutations expand in different cells in cases with multiple mtDNA deletions.^24^ Therefore, to explore mtDNA it is essential to focus on single cells, particularly when investigating pathogenicity of mtDNA mutations.

In this study, we explored mitochondrial respiratory chain activity (complex IV and complex II) histochemically and mtDNA within single neurons in cerebral cortex and immediate subcortical white matter (WM) from SPMS cases. We propose that bouts of acute inflammation and diffuse chronic inflammation in MS damage mtDNA and, through repair processes and clonal expansion, give rise to high heteroplasmy levels of mtDNA deletions in single cells and respiratory-deficient neurons.

## Materials and Methods

### Autopsy Tissue

A total of 98 blocks (approximately 2.5 cm^3^) from 13 SPMS cases and 76 blocks from controls were used in this investigation. MS and control frozen blocks, stored at −80°C, were obtained from the MS Society Tissue Resource, London (Table 1). Control cases had no known neurological disorder or pathology and the age range was between 60 and 88 years. Mean age of MS cases (54.9 ± 13.5 years) was significantly lower than controls (73.8 ± 8.8 years, *p* = 0.001). The postmortem delay in hours between MS cases (11.8 ± 4.8 hours) and controls (16.1 ± 7.2 hours) was not significantly different (*p* = 0.103). Clinical details were obtained from case reports, made available by MS Society Tissue Resource. The Newcastle and North Tyneside Local Research Ethics Committee approved the study.

**Table:** Clinical Details of All Cases Used in This Investigation

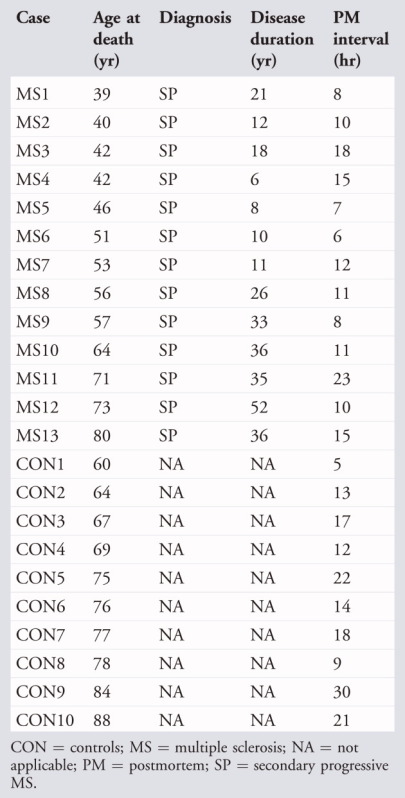

### Immunohistochemistry

Cryostat sections (15μm thick) were allowed to air dry for 90 min. Sections were fixed in 4% paraformaldehyde at 4°C and briefly washed. Antigen retrieval was carried out by boiling sections in 1mM EDTA pH 8 for 1 min; 3% H_2_O_2_ was used to block endogenous peroxidases. Primary antibodies were diluted to the optimized concentrations and applied for 90 min (Supplementary Table S1). Sections were developed using either chromogens (DAB and Vector® SG) or secondary antibody directly conjugated to rhodamine (Jackson Immunoresearch), as previously described.^8^ Appropriate stainings were carried out to exclude cross-reactivity and nonspecific binding of antibodies.

Characterization of the GM lesions was based on previous work.^25^ Both human leukocyte antigen (HLA) and leukocyte common antigen (LCA) antibodies were used to detect inflammation in relation to lesions. Type I or leukocortical lesions span GM and WM, type II lesions are intracortical, often surrounding a blood vessel, and type III or subpial cortical lesions extend from the pial surface partially through the cortex and sparing the deeper layers.^25^, ^26^

### Complex IV/Complex II Histochemistry

Mitochondrial respiratory chain activity was determined using sequential complex IV(COX)/complex II (succinate dehydrogenase [SDH]) histochemistry. Complex IV activity was detected using COX medium.^15^ Sections were washed twice in PBS and the SDH medium, an indicator of complex II activity, was applied.^15^ Sections were dehydrated in 70%, 95%, and 100% ethanol and mounted in an aqueous medium. To identify neurons, NeuN was immunofluorescently labeled following COX/SDH histochemistry. The sections were then prepared as for immunohistochemistry.

### Microscopy and Quantitation

A Zeiss Axioplan microscope was used to obtain brightfield and fluorescent images. All high-magnification images were taken at ×100 with an oil lens. To determine respiratory-deficient neuron density, total GM, and immediate subcortical WM—identified as the WM region bordering and within 250μm from layer VI—areas were calculated separately by scanning the entire sections at low magnification and measuring regions of interest using the AxioVision 4.8 program. Neurons with a NeuN- labeled nucleus were included in the quantitation. Respiratory-deficient neurons were mapped in subcortical and cortical regions twice by 2 investigators who were blinded to the distribution of lesions and type of case.

For neuronal counts, GM and subcortical regions were imaged at ×10 magnification (Zeiss Axioplan) in both MS cases and controls. Cells with NeuN-positive nuclei and with NeuN staining in the cytoplasm, frequently observed, were then recorded using Image Pro®. For an unbiased quantitation, NeuN-positive cells at least 10μm in diameter and with a nucleus were included by setting a threshold. Maps of cortical and WM lesions were made and superimposed to determine the relation of respiratory deficiency to lesions. Quantitation was performed by an individual blinded to case details and lesion location.

Inflammatory cell counts were based on the number of HLA-positive cells in a ×10 magnification field (4 fields per region). Type I lesions were divided into WM and GM components. The association of inflammatory cells to respiratory- deficient cells was explored using brightfield combined with immunofluorescent histochemistry images using a Zeiss AxioImager2 at ×63.

### Terminal Deoxynucleotidyl Transferase- Mediated dUDP Nick-End Labeling

Terminal deoxynucleotidyl transferase-mediated dUDP nick-end labeling (TUNEL; Roche) was carried out per manufacturer's guidelines following COX/SDH histochemistry. Cells were identified at ×100 magnification.

### Laser Microdissection and DNA Extraction

For laser microdissection, cryostat sections (20μm thick) were mounted onto membrane slides (Leica) and histochemistry was performed. Tissue (approximately 250 × 250 μm^2^) was dissected from normal-appearing GM (NAGM), normal-appearing immediate subcortical WM and GM lesions (type I and type III) separately in several serial sections using a Leica laser microdissection microscope (Leica LMD). To determine mtDNA deletions at a single-cell level, both respiratory-efficient (intact complex IV activity) and respiratory-deficient (devoid of complex IV and with complex II activity) neurons were microdissected from GM and immediate subcortical WM. DNA extraction was carried out using the QIAamp DNA Micro Kit (Qiagen) per manufacturer's guidelines.

### Long-Range PCR

Long-range PCR, a well-established technique in diagnostic mitochondrial laboratories, was used to detect mtDNA deletions irrespective of the heteroplasmy level in MS cases and controls. PCR reactions were performed using the Expand Long Template PCR System (Roche) on extracted DNA from 250 × 250-μm^2^ regions and pooled single neurons. To obtain a satisfactory amount of DNA for long range, 5 or 2 neurons were pooled at a time. The following conditions were used for the PCR system: 3 min at 93°C; 10 cycles of 93°C for 30 sec, 58°C for 30 sec, and 68°C for 12 min; 20 cycles of 93°C for 30 sec, 58°C for 30 sec, and 68°C for 12 min plus 5 sec per additional cycle; and a final extension of 11 min at 68°C. The product was diluted 1:30 and 1μl used as a template for a second round using the same conditions as above. Primers used for the study covered the majority of the major arc (Supplementary Fig S1). Long-range products were separated using a 0.7% agarose gel containing ethidium bromide and viewed under UV light. Wild-type DNA from control blood was used as a positive control.

### Gel Extraction and Sequencing

To confirm mtDNA deletions in neurons, amplified products were extracted using the Qiagen gel extraction kit per the manufacturer's instructions. The final concentration of DNA was determined using a spectrophotometer (Nanodrop®). A total of 2μl of ExoSAP was added to each sample while kept on ice. Samples were then incubated for 15 min at 37°C and 15 min at 80°. Cycle sequencing was set up on the samples using the following master mix constituents: deionized water, 5× sequencing buffer, universal forward and reverse primers, and Big Dye v3.1. The cycle sequencing program consisted of 1 min at 96°C and 25 cycles of 10 sec at 96°C, 5 sec at 50°C, and 4 min at 60°C. Samples were sequenced on an ABI 3100 Genetic Analyzer (Applied Biosystems) and compared to the revised Cambridge reference sequence (rCRS) with SeqScape software (Applied Biosystems).

### Real-Time PCR

Real-time PCR, a technique designed to investigate heteroplasmy level rather than as a tool to rule out the presence of mtDNA deletions, was used for the analysis of deletion levels in COX-positive and COX-negative neurons. The multiplex real-time *MTND1/MTND4* assay was used to quantify levels of mtDNA deletions in neurons.^18^, ^22^, ^27^, ^28^ The assay provides a deletion (heteroplasmy) level in the neurons. A total of 5μl of sample was used and run in triplicate in a 96-well plate. A 24-μl master mix consisted of deionized water, TaqMan® Universal mastermix (Applied Biosystems), 100nM ND1 VIC probe, 100nM ND4 FAM probe, 300nM ND1 FOR, 300nM ND1 REV, 300nM ND4 FOR, and 300nM ND4 REV primers (Eurofins). The PCR program consisted of a 2-min incubation at 50°C, 10 min at 95°C and 40 cycles of amplification; 15 sec denaturation at 95°C; and 1 min at 60°C for hybridization of probes and primers and DNA synthesis. Known deletion-level standards, a blood-positive control and a blood-negative control, all run in triplicate, were added to the assays as previously described.^27^

### Statistics

SPSS version 17 was used for statistical analysis. Nonparametric tests (Kruskal-Wallis test for differences between more than 2 groups and Wilcoxon-Mann-Whitney U-test for differences between 2 groups) were used given the uneven distribution of data. A *p* value of <0.05 was considered significant.

## Results

### Respiratory-Deficient Neurons Are Present in Excess of Age in MS Cases

**Figure 1:**
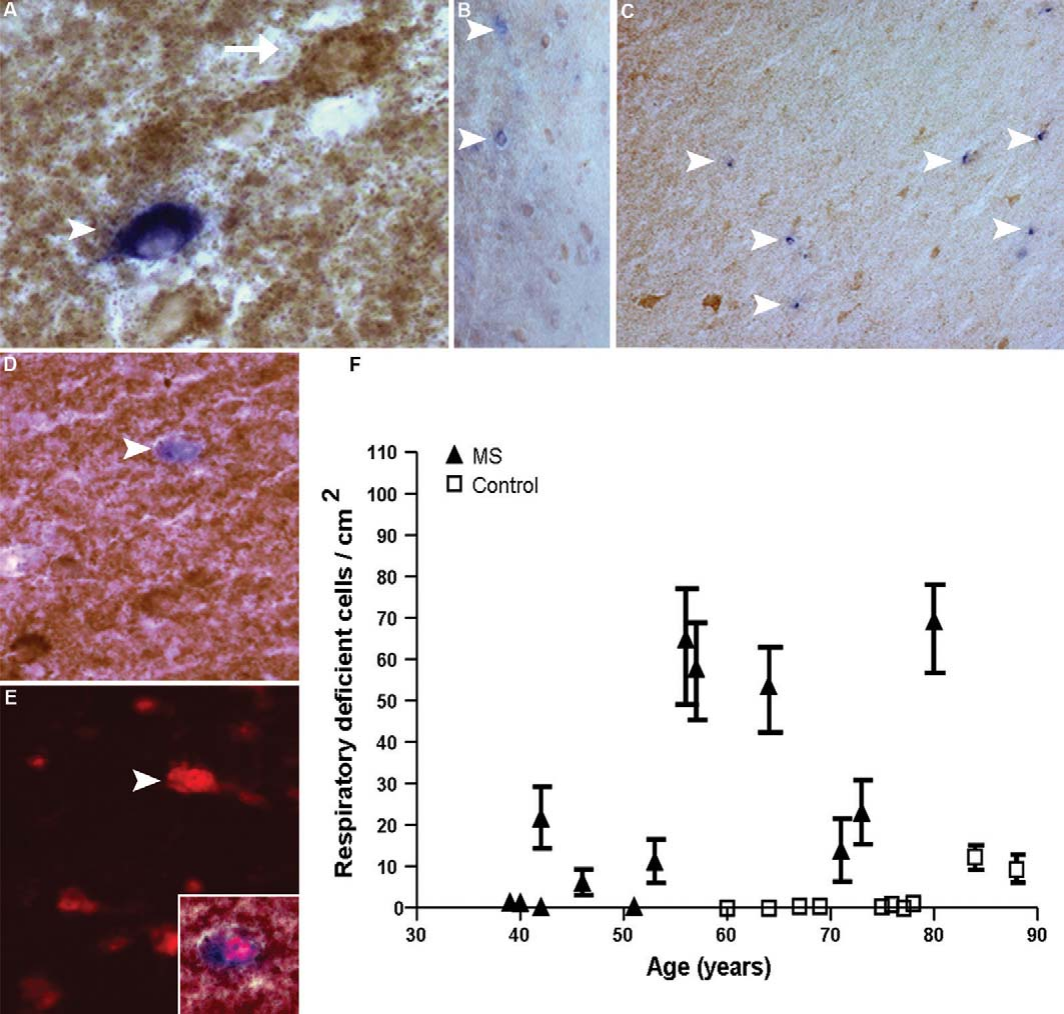
Complex IV (COX)/complex II (SDH) histochemical detection of respiratory-deficient neurons in snap frozen MS tissue sections. Complex IV (COX)/complex II (SDH) histochemistry on snap frozen sections from cases with secondary progressive MS showed respiratory-deficient neurons (lacking complex IV and with complex II stained *blue*, *arrowheads*) as well as neurons with intact complex IV activity (stained *brown*, *arrow*)(A, B) in the GM, (B) predominantly in layer VI, and (C) immediate subcortical WM. Based on morphology both pyramidal and nonpyramidal neurons showed the mitochondrial defect. The majority of respiratory-deficient neurons were located in layer VI (45%) and immediate subcortical white matter (34%). (D, E) Respiratory-deficient cells in GM and WM were confirmed as neurons judged by NeuN immunofluorescent labeling (inset shows respiratory-deficient neuron indicated by *arrowhead*). (F) The average density of complex IV–deficient neurons, from a median of 7 blocks containing frontal, parietal, temporal, and occipital GM and immediate subcortical WM per case, is shown in relation to age for MS cases (▴) and controls (□). The average density represents respiratory-deficient neurons in whole GM and immediate subcortical WM. The mean density of respiratory-deficient neurons in MS cases was significantly greater (24.97 ± 4.67 neurons/cm^2^, n = 13) than aged control brains (1.59 ± 0.86 neurons/cm^2^, n = 10, *p*= 0.003). On average 18.54cm^2^ of GM and 2.26cm^2^ of immediate subcortical WM regions were examined per case. The mean age of MS cases (54.9 ± 13.5) was significantly lower than controls (73.8 ± 8.8, *p*= 0.001). The postmortem delay between MS cases (11.8 ± 4.8 hours) and controls (16.1 ± 7.2 hours) was not significantly different (*p*= 0.103). Differences between 2 groups were analyzed using the Wilcoxon-Mann-Whitney U-test. Error bars indicate standard error of the mean for each case; ± values indicate standard deviation. COX = cytochrome c oxidase or complex IV; GM = gray matter; MS = multiple sclerosis; SDH = succinate dehydrogenase or complex II; WM = white matter. matter. [Color figure can be viewed in the online issue, which is available at www.annalsofneurology.org.]

To identify respiratory-deficient neurons most likely to harbor mtDNA deletions we used frozen tissue sections, COX/SDH histochemistry, and immunofluorescent histochemistry.^12^, ^18^, ^28^ The cells devoid of complex IV activity and with complex II (respiratory-deficient) are stained blue by the histochemical technique as opposed to the cells with complex IV activity, which are stained brown (Fig 1A–C). Following COX/SDH histochemistry, respiratory-deficient neurons were identified by immunofluorescent labeling of NeuN (see Fig 1D, E). A comprehensive analysis of 13 SPMS cases (see Table 1) with age ranging from 39 to 80 years showed a significantly greater density of respiratory-deficient neurons (blue) in MS compared with controls (age ranging from 60 to 88 years), despite MS cases being significantly younger than controls (see Table 1). The majority of respiratory-deficient neurons were located in cortical layer VI and immediate subcortical WM of MS tissue. TUNEL-positive nuclei, caspase-9 expression, or nuclear translocation of apoptosis inducing factor were not detected in respiratory-deficient neurons (not shown). Moreover these neurons were not in close contact with microglia (Supplementary Fig S2). Interestingly, not all SPMS cases showed respiratory-deficient neurons.

### Respiratory-Deficient Neurons Are Globally Distributed and Not Restricted to Lesions

Of the 98 snap-frozen blocks from the MS cases, 81 contained 1 or more of the 3 types of cortical lesions, 96 lesions in total.^25^, ^26^ Type I or leukocortical lesions accounted for 39%(37/96), type II lesions accounted for 10%(11/96), and type III or subpial lesions accounted for 50%(48/96) of the 96 cortical MS lesions. In terms of WM lesions there were 17 in the 98 blocks, with 5 extending up to but sparing the cortex, as described by Chang et al.^29^

**Figure 2:**
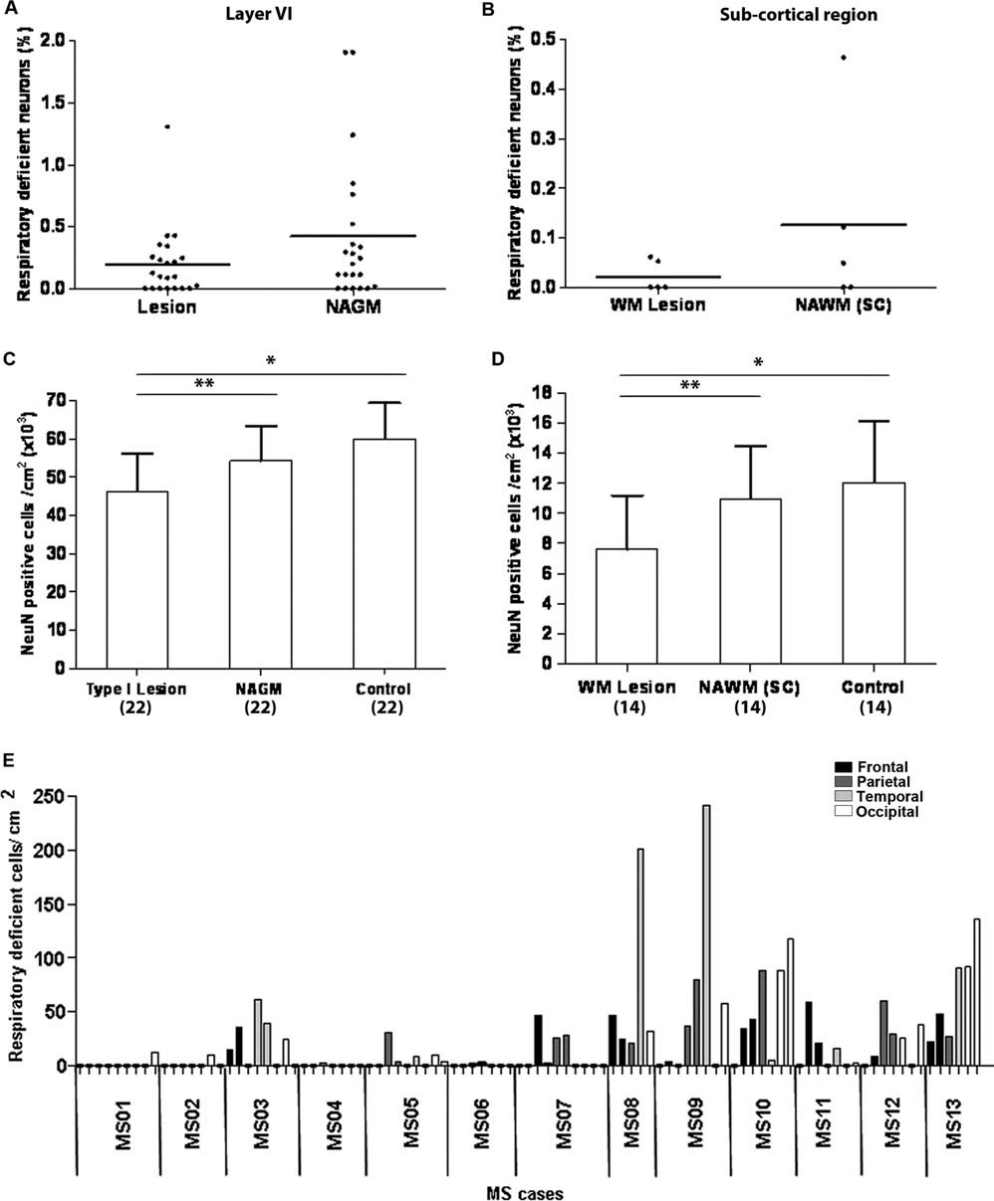
Prevalence of respiratory-deficient neurons in relation to lesions in MS cases. Layer VI neurons in (A) leukocortical (type I) lesions and (C) corresponding NAGM. (B, D) Immediate subcortical WM neurons within WM lesions extending up to the cortex and corresponding NAWM areas [NAWM (SC)]. In A and B, respiratory-deficient neurons, as a percentage of all NeuN-positive cells identified in serial sections, were not significantly different in lesion areas of layer VI (2.01 ± 0.31 out of 1000 neurons in randomly selected type I lesions, n = 22) and lesion areas of immediate subcortical WM (0.23 ± 0.31 out of 1000 neurons, n = 5) compared with corresponding NAGM (4.25 ± 0.41 out of 1000 neurons, n = 22) or WM (1.3 ± 1.9 out of 1000 neurons, n = 5) areas. There was a trend toward more respiratory-deficient neurons in normal-appearing tissue. In C and D, neuronal loss in leukocortical (type I) lesions (in C, 461.49 neurons/mm^2^ ± 97.91) and WM lesions extending up to the cortical margin (in D, 75.76 neurons/mm^2^ ± 36.15) was significant compared to corresponding normal-appearing tissue (GM: 541.93 neurons/mm^2^ ± 91.19; WM: 109.64 neurons/mm^2^ ± 34.84). Neuronal loss in lesion areas was also significant when compared with corresponding areas in control cases (GM: 599.34 neurons/mm^2^± 93.45; WM: 120.52 neurons/mm^2^ ± 40.67). The density of NeuN-positive cells in normal-appearing tissue was not significantly different compared with corresponding regions in controls. In C and D, *parentheses* indicate the number of regions quantitated. (E) When the density of respiratory-deficient neurons in GM and immediate subcortical WM in MS were determined with respect to location (frontal, parietal, temporal, and occipital lobes, and plotted in order with frontal blocks to the left and occipital blocks to the right for each case), the density of respiratory-deficient neurons did not significantly differ between the 4 lobes. Differences between 2 groups (in A and B) and more than 2 groups (in C–E) were analyzed using the Wilcoxon-Mann-Whitney U-test and Kruskal-Wallis test, respectively. *p = 0.005, **p = 0.027, and **p = 0.018 for GM and WM, respectively. Error bars and ± indicate standard deviation. GM = gray matter; NAGM = normal appearing gray matter; NAWM (SC)= normal appearing white matter (immediate subcortical region); MS = multiple sclerosis; WM = white matter.

We determined the prevalence of respiratory-deficient neurons as a percentage of NeuN-positive cells using adjacent sections, to account for neuronal loss. Cortical layer VI and immediate subcortical WM were considered because the majority of respiratory-deficient neurons were located in these regions. Layer VI of leukocortical (type I) lesions and WM lesions extending up to the cortex were compared with the corresponding normal-appearing white matter (NAWM) and NAGM in MS cases and controls (Fig 2A–D). The density of NeuN-positive cells was significantly lower in layer VI of leukocortical (type I) lesions compared with NAGM in MS and controls (see Fig 2C). A significant decrease in neurons was also detected in the immediate subcortical WM of MS lesions compared with corresponding NAWM and regions in controls (see Fig 2D). When corrected for neuronal loss, the percentage of respiratory-deficient neurons in layer VI of type I lesions and immediate subcortical WM of WM lesions extending up to the cortex did not significantly differ when compared with corresponding normal-appearing tissue regions. Interestingly, there was a trend toward more respiratory-deficient neurons in normal-appearing tissue compared with lesions. We then compared the density of respiratory-deficient neurons in MS based on location (frontal, parietal, temporal, and occipital lobes). The difference in the percent of respiratory-deficient neurons between the 4 locations was not significant (see Fig 2E).

### Mitochondrial DNA Deletions Are Widespread in MS Cortex

**Figure 3:**
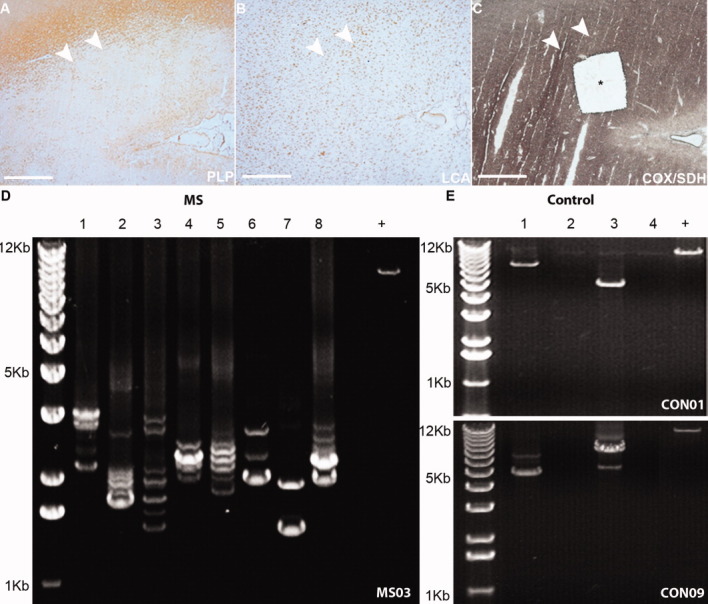
mtDNA deletions are present throughout the GM in MS cases. A cortical MS lesion (*arrowheads*) is identified by (A) lack of PLP and (B) presence of LCA staining. (C) Complex IV (COX)/complex II (SDH) histochemistry was performed in adjacent sections mounted on membrane slides (Leica). In C, approximately 250 × 250-μm^2^ regions from lesions (*) and NAGM were laser-microdissected. Scale bar: 250μm. Long-range PCR using DNA extracted from GM regions from (D) MS cases (shows findings in MS03, aged 42 years) and (E) controls (shows findings in CON01 and CON09, aged 60 and 84 years, respectively) show evidence of multiple deletions of mtDNA (amplified fragments smaller than 11kb). In D, MS cases, mtDNA deletions were extensive in cortical lesions (*lanes 1–4*) and in NAGM (*lanes 5–8*). In E, aged controls, mtDNA deletions were less apparent in cortical GM (*all lanes*) and only the wild-type bands (faint band at 11kb) were observed in a proportion of sampled regions (*lanes 2 and 4*). In E, the mtDNA deletions in controls also vary in size consistent with multiple deletions of mtDNA as evident in the lanes of cases CON1 and CON09. *+* indicates wild-type 11-kb band amplified from control DNA. Scale bar: 500μm. COX = cytochrome c oxidase or complex IV; GM = gray matter; LCA = leukocyte common antigen; NAGM = normal appearing gray matter; MS = muscular sclerosis; PLP = proteolipid protein; SDH = succinate dehydrogenase or complex II; WM = white matter. matter. [Color figure can be viewed in the online issue, which is available at www.annalsofneurology.org.]

We laser-microdissected regions (approximately 250 ×250μm^2^) from NAGM (all MS cases), cortical MS lesions (4 MS cases), and GM from controls (4 cases) and then extracted DNA for long-range PCR (Fig 3A–C) to determine whether mtDNA deletions are present in MS tissue. Multiple deletions of mtDNA were evident in 66% of the 98 NAGM regions analyzed (see Fig 3D). Deletions were also evident in 53% of the 32 lesions analyzed (51% in type I and 55% in type III). In comparison, only 16% of 37 analyzed regions from 4 aged controls showed evidence of mtDNA deletions (see Fig 3E). Interestingly, we did not find evidence of mtDNA deletions by long-range PCR in 2 MS cases (MS02 and MS07).

### mtDNA Deletions Are Present Within Neurons

**Figure 4:**
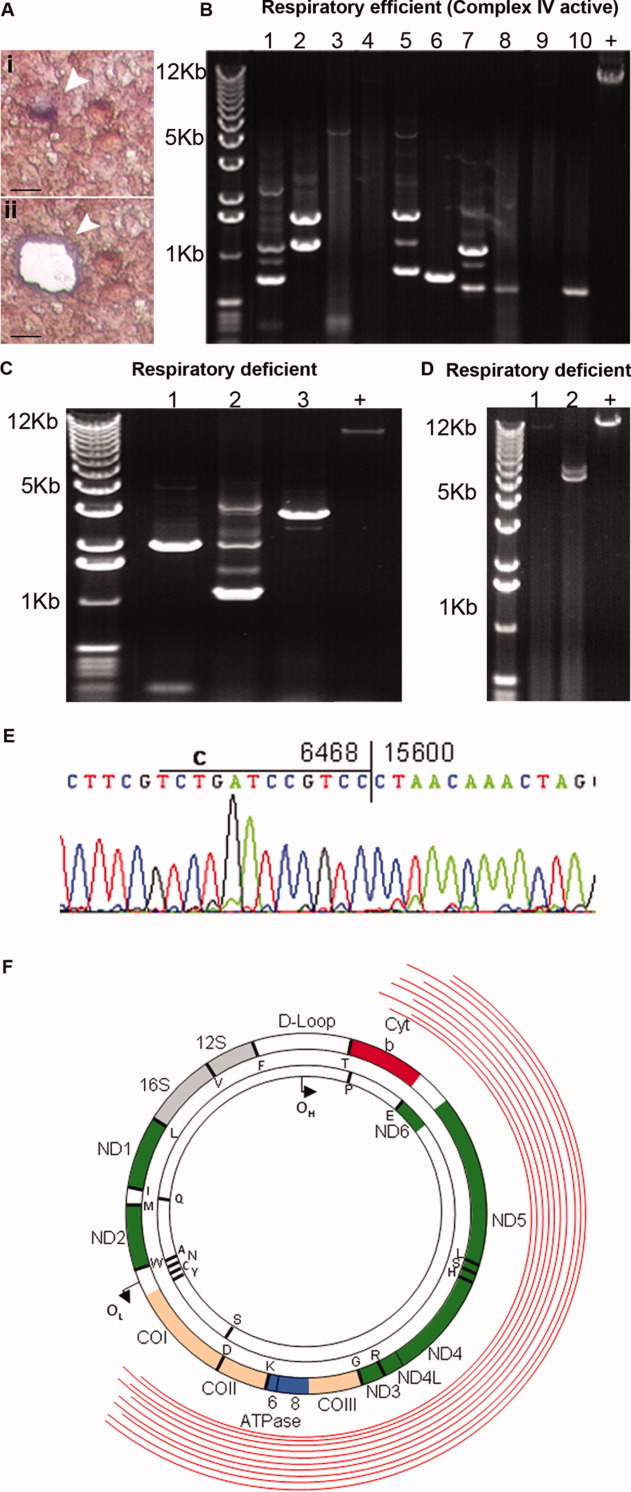
mtDNA deletions are present within neurons in MS. (A) Following complex IV (COX)/complex II (SDH) histochemistry and immunofluorescent labeling of NeuN, single respiratory-deficient neurons (i, *arrowhead*) and neurons with complex IV activity (*brown*) were isolated from membrane slides using laser microdissection (ii). Scale bar: 20 μm. (B) Long-range PCR identified multiple deletions of mtDNA in respiratory efficient neurons (*brown*) when DNA was extracted from 5 pooled, closely located neurons (shown in each lane). mtDNA deletions were evident within neurons throughout the MS GM (MS03 shown with each lane representing different regions sampled from frontal, parietal, temporal, and occipital lobes). When (C) 5 and (D) 2 respiratory-deficient neurons from MS cases were pooled there was also evidence of mtDNA deletions. In D, the minimum number of neurons needed to extract adequate quantity of mtDNA for long-range PCR was 2. In C and D, the number of mtDNA deletions detected by long-range PCR rarely exceeded the number of respiratory-deficient neurons pooled, consistent with clonal expansion. As previously reported, the full-length product is not always apparent, particularly when mtDNA deletions are present possibly due to bias of PCR.^28^, ^31^ (E, F) Sequencing of the deleted mtDNA extracted from long-range PCR gels identified the breakpoints of deletions, as shown in the electropherogram, confirming both long-range and real-time PCR findings. (G) The large-scale deletions encompassed much of the major arc of the mitochondrial genome including genes of complex I (*green*), complex III (*red*), complex IV (*orange*), complex V (*blue*), and tRNAs (*black*). Scale bar: 10μm. + indicates wild-type 11kb band amplified from control DNA. COX = cytochrome c oxidase or complex IV; GM = gray matter; MS = muscular sclerosis; SDH = succinate dehydrogenase or complex II. [Color figure can be viewed in the online issue, which is available at www.annalsofneurology.org.]

Given the likelihood that respiratory-deficient neurons lacking complex IV and with complex II activity harbor mtDNA deletions, we explored mtDNA extracted from pooled laser-microdissected single neurons based on morphology and NeuN labeling (Fig 4A). Long-range PCR using DNA extracted from 5 pooled neurons with complex IV activity per lane identified multiple deletions of mtDNA in these respiratory-efficient neurons (see Fig 4B). MtDNA deletions were also present in 5 pooled respiratory-deficient neurons (see Fig 4C). In DNA pooled from 2 respiratory-deficient neurons, the minimum number needed to extract a sufficient quantity of mtDNA for long-range PCR, there were multiple deletions of mtDNA (see Fig 4D). However, the number of mtDNA deletions rarely exceeded the number of pooled neurons in long-range PCR (see Fig 4C, D). Sequencing the deleted mtDNA extracted from the long-range PCR product verified the presence of mtDNA deletions and characterized the breakpoints (see Fig 4E). All sequenced mtDNA deletions confirmed removal of genes encoding key subunits of respiratory chain complexes, except complex II, which is entirely encoded by nuclear DNA, and tRNAs (see Fig 4F). Nearly all deletions involved imperfect repeat sequences similar to previous findings within neurons (Supplementary Table S2).^30^

### mtDNA Deletions Are Expanded to High Heteroplasmy Levels in Single Respiratory-Deficient Neurons

**Figure 5:**
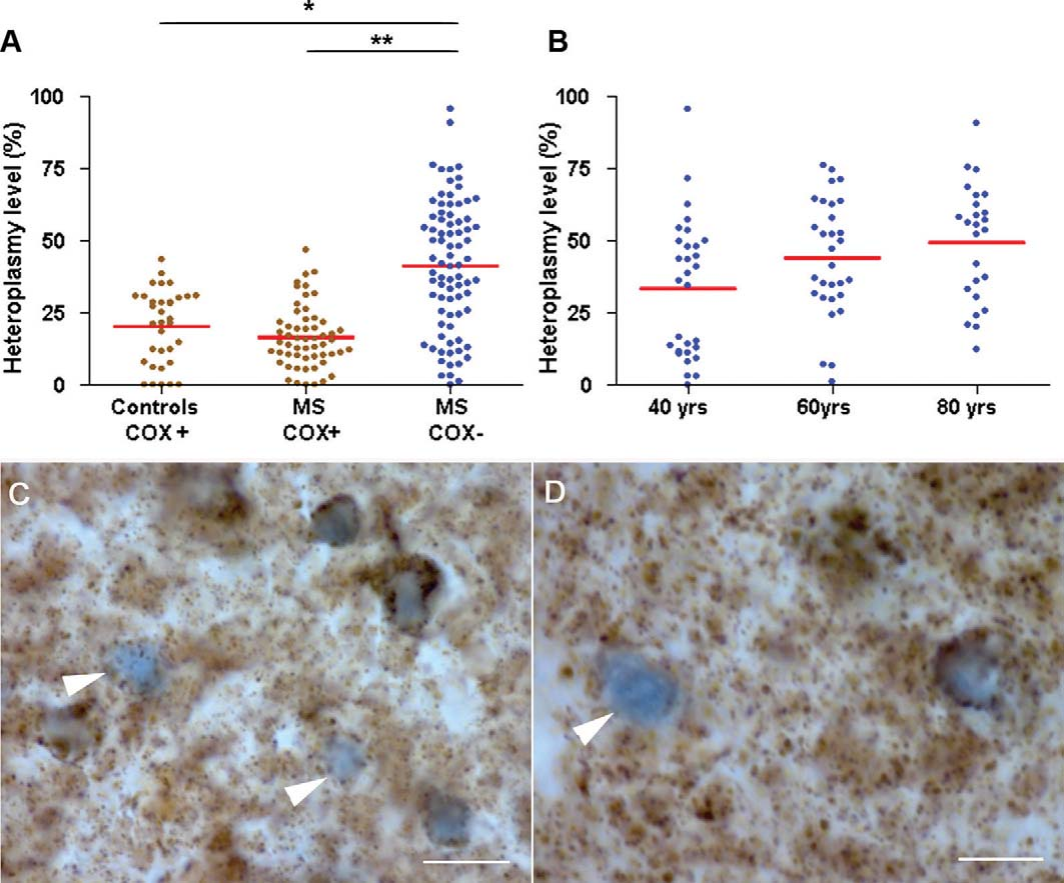
High heteroplasmy levels of mtDNA deletions in single respiratory-deficient neurons. (A) The heteroplasmy level of mtDNA deletions at a single-cell level was determined using real-time PCR and DNA extracted from single neurons; respiratory-deficient (*blue*) neurons contained significantly greater levels of mtDNA deletions (41.22%± 22.64), judged by ND1 and ND4 assay, compared with those with intact complex IV activity (*brown*) in MS cases (16.43%± 10.72, **p < 0.001) and controls (20.31%± 13.71, *p= 0.001). Based on 95% confidence intervals of heteroplasmy level in respiratory-efficient neurons (*brown*) from MS cases (n = 6) and controls (n = 5), 55% of respiratory-deficient neurons in MS cases had the increased heteroplasmy levels. We did not find a correlation between heteroplasmy level and density of HLA-positive cells. Both GM and WM neurons were analyzed (53% and 47%, respectively of all neurons analyzed). In total, 34, 56, and 80 single neurons were included for control COX+, MS COX+, and MS COX− neurons. (B) High heteroplasmy levels at a single neuronal level were detected in respiratory-deficient neurons in MS cases, independent of age. Immunohistochemical labeling of (C) COX-I and (D) COX-II in adjacent sections using DAB (*brown*), both of which are mtDNA-encoded catalytic subunits of complex IV, and NeuN (Vector® SG, *blue*) identify neurons lacking mtDNA-encoded subunits (*arrowheads*). Scale bar: 16 μm. Kruskal-Wallis test was used to compare difference between groups. COX+= respiratory-efficient neurons with intact complex IV activity (brown histochemical stain); COX−= respiratory-deficient neurons devoid of complex IV and with complex II activity; GM = gray matter; HLA = human leukocyte antigen; MS = multiple sclerosis; WM = white matter. [Color figure can be viewed in the online issue, which is available at www.annalsofneurology.org.]

In order to investigate the pathogenicity of mtDNA deletions within neurons we determined the heteroplasmy level of mtDNA deletions at a single-cell level in respiratory- deficient neurons and compared with the levels in neurons with intact complex IV activity (Fig 5A). The mean heteroplasmy level of mtDNA deletions in single respiratory-deficient neurons from 6 MS cases was significantly greater than in neurons with intact complex IV activity from MS cases and neurons from control cases (see Fig 5A). Based on 95% confidence interval of heteroplasmy in respiratory-efficient neurons, 55% of respiratory- deficient neurons in MS contained significantly higher heteroplasmy levels of mtDNA deletions. Neurons with high heteroplasmy levels of mtDNA deletions were found in young and aged MS cases (see Fig 5B). We did not detect a significant difference in heteroplasmy levels between the WM and GM components of type I lesions. The pathogenicity of mtDNA deletions was further confirmed by the loss of catalytic subunits of complex IV (COX-I and COX-II), which are encoded by mtDNA, and genes for which are often absent in deleted mtDNA (see Fig 5C, D).

## Discussion

In this study we present evidence for the first time that multiple deletions of mtDNA cause respiratory deficiency within neurons as a result of clonal expansion in MS. There were strikingly more neurons lacking complex IV and with complex II activity (respiratory-deficient) in SPMS cases compared with aged controls. The respiratory-deficient neurons were distributed diffusely and found irrespective of lesions. Our findings identify respiratory deficiency due to mtDNA deletions as a potentially important pathomechanism in MS.

Clonally expanded multiple deletions of mtDNA causing respiratory deficiency are well recognized in neurodegenerative disorders and aging.^18^, ^21^, ^24^, ^31^ Despite the significantly younger age of MS cases the respiratory-deficient neurons were more prevalent compared with aged controls in this study. Given the vulnerability of mtDNA to oxidative damage and the extent of inflammation in MS, often starting with a preclinical phase and remaining throughout the disease course, mtDNA deletions might be expected in MS.^19^, ^20^ Repair of damaged mtDNA rather than oxidative damage to mtDNA per se is the most likely mechanism by which mtDNA deletions are formed, and clonal expansion is regarded as the mechanism responsible for causing respiratory deficiency.^20^, ^21^, ^24^ Where respiratory deficiency is caused by induced multiple DNA deletions, cells will have initially contained deletions with different breakpoints, one of which then clonally expands to high levels over time.^23^, ^24^ Clonally expanded multiple deletions of mtDNA are reported in inclusion body myositis, a condition associated with chronic inflammation.^32^, ^33^ Our long-range and real-time PCR findings within respiratory-deficient neurons from SPMS cases are consistent with clonally expanded multiple deletions of mtDNA. Study of mtDNA in other forms of MS would be of interest. Our prediction in relapsing remitting stage of MS (RRMS) is that inflammation would still induce mtDNA deletions. However, mtDNA deletions may not have had adequate time to expand to threshold levels to cause respiratory deficiency in RRMS, in which disease duration is shorter than in SPMS. Whether mtDNA deletions and clonal expansion play a role in the primary progressive form of MS needs investigation.

We did not find an increase in density of respiratory-deficient neurons in MS lesions where inflammation is most prominent. In fact there was a trend toward a decrease in respiratory-deficient neurons in lesions. Respiratory-deficient neurons may be less capable of withstanding an inflammatory environment. In lesions, neurons are exposed to a number of insults and mitochondria may dysfunction as a result of not only mtDNA deletions but also direct inhibition by nitric oxide, modification and inactivation of subunits by peroxynitrite, and degradation of mRNA by ROS.^34–36^ In environments with much less inflammation than MS, clonally expanded mtDNA deletions cause neurodegeneration.^37^ Lack of TUNEL-positive nuclei, caspase expression, and nuclear translocation of AIF in respiratory-deficient neurons in MS and primary mitochondrial disorders suggests the need for another local factor or “second hit” before undergoing cell death.^37^ The slow rate of global neuronal loss in chronic neurodegenerative disorders and rapidity of apoptosis make the capture of apoptotic cells in postmortem tissue difficult. In addition to the energy defect, calcium handling is impaired in neurons harboring mtDNA mutations.^38^ Neurons as well as axons and myelin with mitochondrial defects are more susceptible to glutamate-mediated injury, as illustrated in vitro using mitochondrial respiratory chain inhibitors.^8^, ^39^, ^40^ Thus, decrease in density of respiratory-deficient neurons in lesions is a likely reflection of mtDNA deletion-mediated cell loss as well as increase in susceptibility to other insults because of the clonally expanded mtDNA deletions.

The presence of respiratory-deficient cells in frontal, parietal, temporal, and occipital lobes irrespective of lesions is supportive of the hypothesis that clonal expansion of mtDNA is a random event.^41^, ^42^ However, predilection of layer VI for respiratory-deficient neurons harboring clonally expanded mtDNA deletions, despite multiple deletions of mtDNA been present throughout cerebral cortex albeit at low levels, suggests that WM events may influence clonal expansion through soluble factors. Layer VI involvement appears to be selective to MS, as it has not been described with aging or in primary mitochondrial disorders, in which local factors, yet to be identified, appear to trigger cell loss.^37^ Nitric oxide is recognized to play a role in mitochondrial biogenesis and mtDNA replication.^43^ Faster rate of turnover of mtDNA and preferential replication of certain mtDNA deletions may account for the above observation in MS.

The extent of mtDNA deletions identified using long-range PCR on a global scale regardless of cell type, and even in neurons with intact complex IV activity, reflects the potential of cells in MS to become respiratory-deficient through clonal expansion over the course of the disease. By isolating individual neurons at a single time point we identified high levels of multiple mtDNA deletions within respiratory-deficient cells. In contrast, mtDNA deletions detected by long-range PCR within respiratory-efficient neurons in MS and controls were not expanded to high levels. Blokhin et al.^44^ concluded in a recent study that the pathology of MS was not associated with an accumulation of complex IV–negative cells and mitochondrial DNA deletions based on similar findings to ours in respiratory competent cells but, crucially, the authors did not study respiratory-deficient neurons in MS cases. Furthermore, long-range PCR was not used to detect mtDNA deletions in MS cases or controls. Corresponding with our data, the authors did report an increase in respiratory-deficient cells with age in healthy individuals; however, MS cases over the age of 53 years were not included. There are several limitations of the real-time PCR and single-cell analysis of mtDNA deletions.^27^ Not all deletions may involve *ND4* and *ND1*, although rarely, may also be deleted. Isolation of single neurons undoubtedly will result in inclusion of some adjacent respiratory competent tissue, which would dilute deleted mtDNA and hence reduce the heteroplasmy levels. Indeed, studies that report induced multiple deletions of mtDNA show a wide spectrum for the heteroplasmy level in respiratory-deficient cells, similar to findings in this study.^18^, ^27^, ^33^, ^45^ The possibility that some of the respiratory-deficient neurons may harbor clonally-expanded mtDNA point mutations needs consideration.

Respiratory deficiency due to mtDNA deletions is likely to be more extensive than that indicated by available histochemical methods. The mtDNA deletions reported in this study not only remove subunits of complex IV but also complex I, complex V, and tRNAs, all of which will significantly hamper ATP synthesis. Histochemical techniques necessary to detect complex I, complex III, and complex V activity at a single-cell level are not available. It is becoming increasingly clear that the basis of energy deficit in MS is multifactorial.^6^, ^8–10^ With respect to neurons in MS several transcripts of nuclear DNA–encoded subunits of the mitochondrial respiratory chain as well as activity of complex I and complex III were decreased in the NAGM homogenates from frontal and parietal lobes in patients with progressive disease.^6^, ^46^ Oxidative damage to mtDNA rather than mtDNA deletions were reported in WM MS lesions.^10^ Once respiratory-deficient due to clonally-expanded mtDNA deletions, the defect is likely to be irreversible and unresponsive to immunomodulatory therapy, unlike mitochondrial dysfunction due to modification of transcripts or respiratory chain proteins. Our findings suggest that inflammation-mediated damage to mtDNA, mtDNA repair processes, and clonal expansion of mtDNA mutations are potential therapeutic targets particularly relevant to the progressive stage of SPMS.^20^

These findings identify mtDNA deletions, induced by inflammation and clonally expanded at a single-cell level causing respiratory deficiency, as potentially important contributors to the pathogenesis of MS. We propose the combination of increased neuronal vulnerability due to mtDNA deletions and a ‘second hit’ from chronic inflammation as an important basis of neurodegeneration in the progressive stage of MS. Protection of mitochondria and mtDNA in MS may help to reduce the irreversible decline of neurological function.
