# Supplementary figures and images for: Mitochondrial DNA deletions and neurodegeneration in multiple sclerosis
Source: Ann Neurol. 2011 Mar;69(3):481–92. doi: 10.1002/ana.22109 (PMC3580047; doi:10.1002/ana.22109)

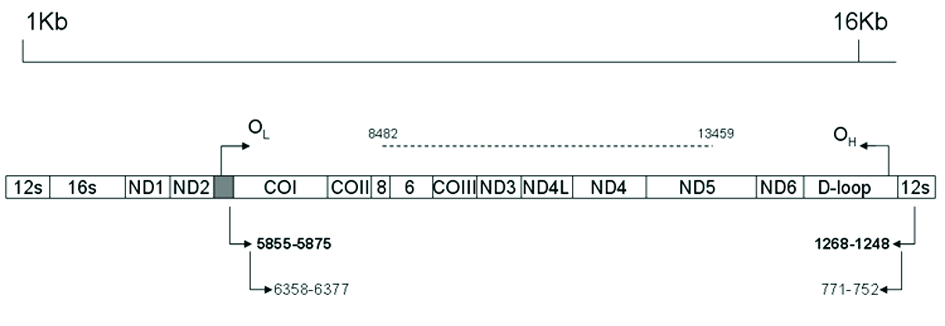

Supplement: Supplementary file 1 [file ana0069-0481-sd1.tif]

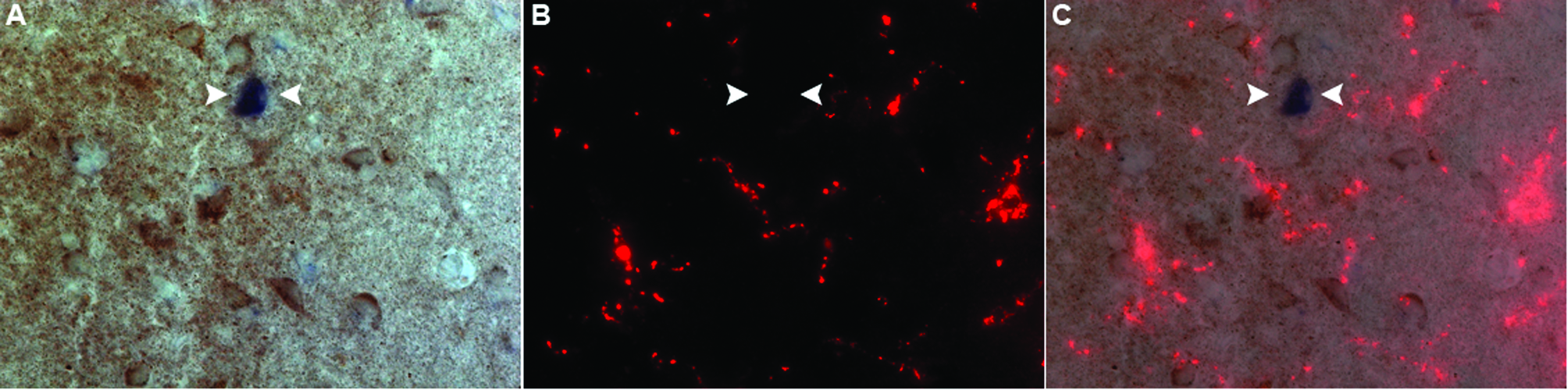

Supplement: Supplementary file 2 [file ana0069-0481-sd2.tif]
