# Supplementary material for: Mitochondrial DNA deletions and neurodegeneration in multiple sclerosis
Source: Ann Neurol. 2011 Mar;69(3):481–92. doi: 10.1002/ana.22109 (PMC3580047; doi:10.1002/ana.22109)
Supplement: Supplementary file 3 [file ana0069-0481-sd3.doc]

**Supplementary Table S1.** Details of antibodies used in this investigation.

| **Antibody** | **Antibody type** | **Target** | **Manufacturer** |
| --- | --- | --- | --- |
| PLP | Mouse IgG_1_  1:1000 | Proteolipid protein | AbD Serotec |
| HLA-DP, DQ, DR | Mouse IgG_1_  1:50 | Human leukocyte antigen | Dako Cytomation |
| LCA | Mouse IgG_1_  1:20 | Leukocyte common antigen | Invitrogen |
| NeuN | Mouse IgG_1_  1:1000 | Neuronal cells | Millipore |
| MBP | Mouse IgG_1_  1:1000 | Myelin basic protein | Covance |
| COXI | Mouse IgG_2a_  1:100 | Complex IV subunit-I | Mitosciences |
| COXII | Mouse IgG_2a_  1:100 | Complex IV  Subunit-II | Mitosciences |
| AIF | Rabbit polyclonal 1:100 | Apoptosis inducing factor | Cell Signaling Technology |
| Cleaved Caspase-9 | Rabbit polyclonal 1:100 | Caspase-9 | Cell Signaling Technology |
