# Supplementary material for: Mitochondrial DNA deletions and neurodegeneration in multiple sclerosis
Source: Ann Neurol. 2011 Mar;69(3):481–92. doi: 10.1002/ana.22109 (PMC3580047; doi:10.1002/ana.22109)
Supplement: Supplementary file 4 [file ana0069-0481-sd4.doc]

**Supplementary Table S2.** The break points of large scale mtDNA deletions in MS cases.

| Complex IV status | Break points | Type |
| --- | --- | --- |
| COX positive | 6627-15859 | 3’ deleted  Imperfect repeat |
| COX positive | 6400-15449 | 5’ deleted  Imperfect repeat |
| COX positive | 6519-16072 | No repeat sequence |
| COX positive | 6468-15600 | 3’ deleted  Imperfect repeat |
| COX positive | 6625-16076 | 3’ deleted  Imperfect repeat |
| COX positive | 6625-16076 | 3’ deleted  Imperfect repeat |
| COX positive | 6569-16076 | 3’ deleted  Imperfect repeat |
| COX positive | 6468-15600 | 3’ deleted  Imperfect repeat |
| COX positive | 6468-15600 | 3’ deleted  Imperfect repeat |
| COX negative | 6471-15603 | 5’ deleted  Imperfect repeat |
| COX negative | 6460-15591 | Perfect repeat |

Supplementary Table 2. The break points of eleven of the largest mtDNA deletions found in MS cases confirms the deletions detected by long range PCR and identify the majority of the break points as associated with repeat sequences which are in the most part imperfect, akin to previous reports of mtDNA deletions within neurons ^30^.

COX +: respiratory efficient neurons with intact complex IV activity (brown histochemical stain). COX -: respiratory deficient neurons devoid of complex IV and with complex II activity (blue histochemical stain).
